# Extended adverse effects of cyclophosphamide on mouse ovarian function

**DOI:** 10.1186/s40360-020-00468-5

**Published:** 2021-01-07

**Authors:** Jihyun Kim, Sooseong You

**Affiliations:** Clinical Medicine Division, Korea Institute of Oriental Medicine, 1672 Yuseongdae-ro, Yuseong-gu, Daejeon, 34054 Republic of Korea

**Keywords:** Cyclophosphamide, Chronic side effect, Ovarian dysfunction, Oocyte, Bioinformatics analysis, Mice

## Abstract

**Purpose:**

Most patients with cancer undergo multiple administrations of anticancer drugs during treatment, resulting in chronic impairment of their reproductive health. As improved treatment options increase cancer survival, it has become increasingly important to address fertility issues in cancer survivors. In this study, we examined the pathophysiological effects of multiple exposures to cyclophosphamide (Cy) on the ovaries of mice and their underlying molecular mechanism.

**Methods:**

Female C57BL/6 mice were intraperitoneally injected with 100 mg/kg Cy six times over 2 weeks; 4 weeks later, the mice were sacrificed and their ovaries, sera, and oocytes were collected for histological observation, measurement of anti-Müllerian hormone levels, and assessment of oocyte quantity and quality in response to hormonal stimulation. Gene expression changes in Cy-treated ovaries were examined by microarray and bioinformatics analyses.

**Results:**

After repeated Cy exposure, the anti-Müllerian hormone level was decreased, and follicle loss and impairments in the quality of oocyte were irreversible. The expression levels of genes involved in folliculogenesis, oogenesis, and zona pellucida glycoprotein transcription displayed sustained alterations in Cy-exposed ovaries even after 4 weeks.

**Conclusion:**

The adverse effects of Cy on ovarian function and oocytes remained even after chemotherapy was complete. Therefore, strategies to prevent ovarian damage or restore ovarian function after treatment are required to safeguard the fertility of young cancer survivors.

## Background

Most patients with ovarian cancer are administered multiple rounds of chemotherapy, the off-target toxicities of which can result in dangerous side effects that must be addressed [1]. Anticancer agents have complex mechanisms of action and their effects depend on their drug type, dose, and therapeutic duration [2]. During cancer treatment, the same drugs are administered every 2–3 weeks for more than four cycles; this repeated exposure can severely affect the quality of life of patients [3]. In female survivors, concerns include early-onset menopause and treatment-related infertility [4].

The need for female survivors to perform family planning is increasing. Care providers recommend that women wait from 6 months to 2 years after finishing chemotherapy before becoming pregnant to avoid adverse effects on the infant [5]. The long-term effects of repeated exposure to anticancer drugs remain unclear; however, most animal studies have used single or short-term exposures to investigate adverse effects on ovarian function and their mechanisms. Reports on the mechanisms of chronic ovarian dysfunction after repeated cancer treatment are lacking.

Cyclophosphamide (Cy) is a widely used alkylating agent that is toxic to both cancer cells and reproductive cells [6, 7]. Cy exposure directly and indirectly leads to apoptosis by inducing DNA damage, suppressing proliferation, and causing mitochondrial dysfunction, resulting in diminished ovarian reserves [8–11]. A previous study showed that potent regulatory factors can persist or prevent acute ovarian toxicity induced by short-term Cy treatment [12]. Studies are needed to understand the molecular mechanism and changes in gene expression under chronic impaired conditions following rigorous Cy treatment. This may help in the prevention of the extended toxic effects of Cy treatment. To address the chronic effects of repeated Cy treatment, we evaluated ovarian function 4 weeks after the cessation of Cy exposure and investigated the molecular mechanisms underlying chronic ovarian damage.

## Methods

### Mice

Experimental animal protocols were approved by the Institutional Animal Care and Use Committee at Korea Institute of Oriental Medicine (19–019, Daejeon, Korea). Eight-week-old female C57BL/6 mice (18–20 g) were obtained from Narabiotech (Pyeongtaek, Korea) and housed under specific pathogen-free conditions. Animals were randomly divided into two groups and administered intraperitoneal injections of saline without (*n* = 12) or with 100 mg/kg Cy (Sigma-Aldrich, St. Louis, USA) six times over 2 weeks (n = 12). The mice were sacrificed 4 weeks after the final Cy injection. This timeframe was selected because it provides sufficient time for newly recruited primordial follicles to complete the preantral period [13]. Blood was collected from the inferior vena cava of mice anesthetized with 1.2% avertin (0.6 mL/mouse, Sigma-Aldrich). Each mouse was euthanized by cervical dislocation to collect ovaries and oocytes after blood collection. The ovaries were removed, weighed, and immediately fixed in 4% paraformaldehyde (Biosesang, Seongnam, Korea).

### Hormonal assessment by enzyme-linked immunosorbent assay (ELISA)

Sera separated from the blood samples were frozen at − 70 °C until analysis. The concentration of anti-Müllerian hormone (AMH) was measured by ELISA (MyBiosource, San Diego, CA, USA) in triplicate according to a standard protocol and the manufacturers’ instructions. The inter-assay coefficient of variation was < 10% and sensitivity was 0.19 ng/mL.

### Histological assessment of ovarian follicles

The whole ovaries were serially sectioned to 5-μm thickness and stained with hematoxylin and eosin. Primordial, primary, secondary, and preovulatory follicles with visible oocytes were counted in every fifth stained section to avoid counting the same follicle twice. The follicle stage was classified as previously described [14, 15]: primordial follicles had a single flat layer of granulosa cells surrounding the oocyte, primary follicles had a single cuboidal granulosa cell layer, secondary follicles had at least two granulosa cell layers and a theca cell layer, and preovulatory follicles had a complete antrum and theca cell layer.

### Assessment of oocyte quality

Cyclophosphamide- or saline-injected mice were superovulated via intraperitoneal injection of 5 IU pregnant mare’s serum gonadotropin (Prospec, Rehovot, Israel), followed by 5 IU human chorionic gonadotropin (hCG, Prospec) at 48 h later. Oocytes were collected 18 h post-hCG injection in preincubated Human Tubal Fluid medium (Irvine scientific, CA, USA). Oocytes were fixed in 4% paraformaldehyde and permeabilized in 0.5% Triton X-100 (Sigma-Aldrich) for 10 min. Oocytes were blocked in phosphate-buffered saline containing 3% bovine serum albumin (Genedepot, Katy, TX, USA), and then incubated with rabbit anti-α-tubulin antibody (1:200, Cell Signaling Technologies, Danvers, MA, USA). Oocytes were mounted with VECTASHIELD Antifade Mounting Medium with 4′6’-diamidino-2-phenylindole (DAPI, Vector Laboratories, Burlingame, CA, USA) to visualize the chromosomes, and observed by fluorescence microscopy (Olympus BX51, Tokyo, Japan). Oocytes with well-organized, bipolar spindles and chromosomes that were tightly aligned at the metaphase plate were scored as normal. Oocyte quality was also evaluated by measuring morphometrical parameters, including the complete oocyte, ooplasm, and perivitelline space (PVS) using NIS-elements BR 4.60.00 software (Nikon, Tokyo, Japan) [16].

### Microarray analysis

Ovaries from Cy- or saline-injected mice were collected, and total RNA was extracted using the RNeasy Mini kit (Qiagen, Hilden, Germany) according to the manufacturer’s instructions. The purity and integrity of the extracted RNA were evaluated using a NanoDrop ND-1000 UV-Vis Spectrophotometer (Thermo Fisher Scientific, Waltham, MA, USA). All samples were of high purity (optical density (OD)_260_/OD_280_ > 2.00) and integrity (RNA integrity number > 7.0). Hybridization on GeneChip Mouse Gene 2.0 ST arrays (Affymetrix) was controlled using GeneChip Command Console Software (AGCC, Affymetrix, Santa Clara, CA, USA). We used Affymetrix Expression Console 1.4 Software for basic data extraction (CEL files) and quality control metrics. A fold change value > 2.0 and a *p-*value < 0.05 were used as thresholds to identify differentially expressed genes (DEGs). Functional annotation of the DEGs was performed using the Database for Annotation, Visualization and Integrated Discovery version 6.8 (https://david.ncifcrf.gov/hom.jsp). Gene ontology (GO) analysis was performed to identify potential functions of DEGs in the biological process, molecular function, and cellular component categories [17].

### Statistical analyses

Data are presented as means ± standard deviation (SD). The statistical significance of differences between the two groups was determined by Student’s *t*-test using GraphPad Prism, version 8.4.0 (GraphPad, Inc., La Jolla, CA, USA). *P* < 0.05 was considered as statistically significant.

## Results

### Impaired physiological conditions endure after cy exposure

**Fig. 1:**
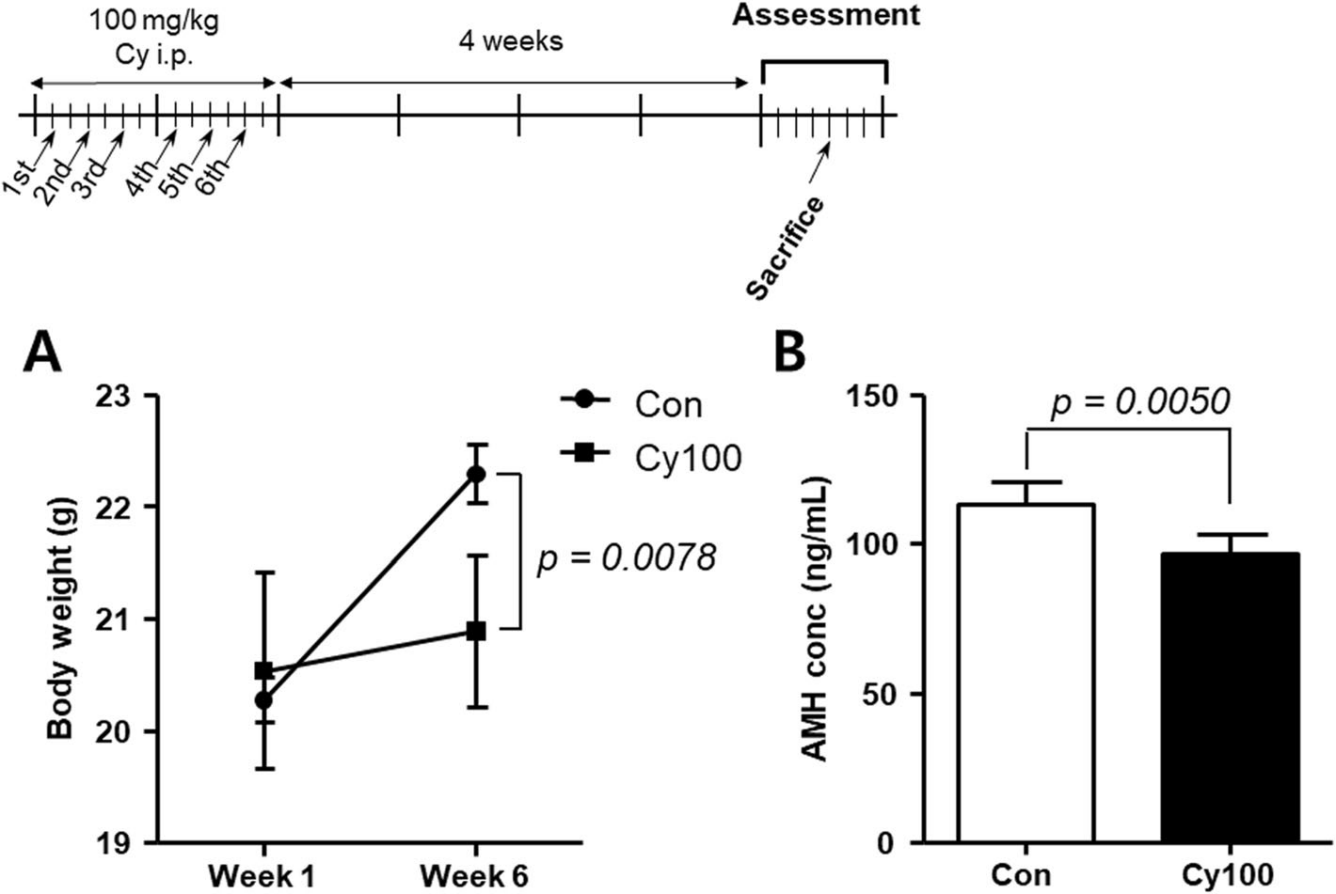
Body weights and serum AMH levels 4 weeks after Cy exposure Mice were injected with Cy (*n* = 5) or saline (*n* = 5) for 2 weeks, and then housed for 4 weeks before the mice and ovaries were weighed, and serum AMH levels were measured. **a** Mouse body weights in individual control and Cy-injected mice. **b** Serum AMH levels in each group. Data represent the mean ± standard deviation. Statistical analysis was performed by Student’s *t*-test

Mice were monitored throughout the study and sacrificed to collect ovaries and blood 4 weeks after Cy treatment. The treated mice had significantly lower body weights than control mice (Fig. 1a). A significant loss of body weight has been associated with negative therapeutic responses [18]. We also measured AMH levels in the serum of Cy- and saline-injected mice (Fig. 1b). The AMH level was significantly decreased in Cy-injected mice, suggesting a decline in the number of growing follicles (*p* < 0.01).

### Follicle loss after cessation of cy exposure

**Fig. 2:**
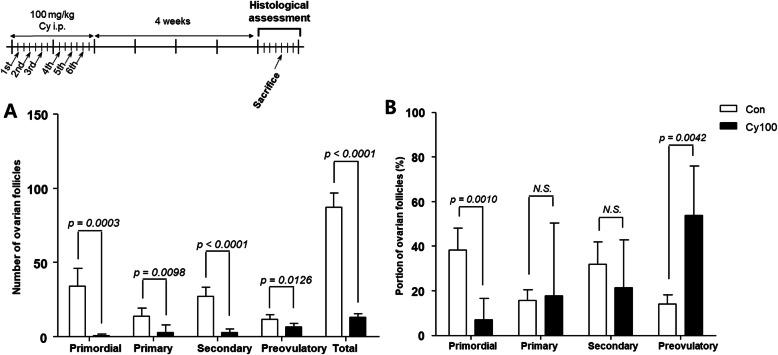
Histological observation of ovarian follicles 4 weeks after Cy exposure Mice were injected with Cy (*n* = 5) or saline (*n* = 5) for 2 weeks, and then housed for 4 weeks before histological observation of the ovaries. **a** Number of ovarian follicles in control and Cy-injected mice. **b** Proportions of primordial, primary, and secondary and preovulatory follicles in each group. Data represent the mean ± standard deviation. Statistical analysis was performed by Student’s *t*-test

To investigate the side effects of rigorous Cy exposure on the ovaries, we performed histological analysis of isolated ovaries 4 weeks after Cy treatment. Entire follicles were damaged, and the number of follicles at all stages was significantly decreased (Fig. 2a, *p* < 0.05). The proportions of primordial and preovulatory follicles were significantly decreased and increased, respectively (Fig. 2b, *p* < 0.01). The decrease in primordial follicles may have been due to a combination of damage and growth activation. Although surviving granulosa cells in the growing follicles secreted AMH, its level was low, and only a small number of growing follicles survived and were activated within one menstrual cycle.

### Cy-induced impairment of oocyte quantity and quality is irreversible

To investigate the effects of rigorous Cy exposure on oocytes, mice were hormonally superovulated. Oocytes were collected from the oviducts 18 h post-hCG, and their quantity and quality were assessed.

**Fig. 3:**
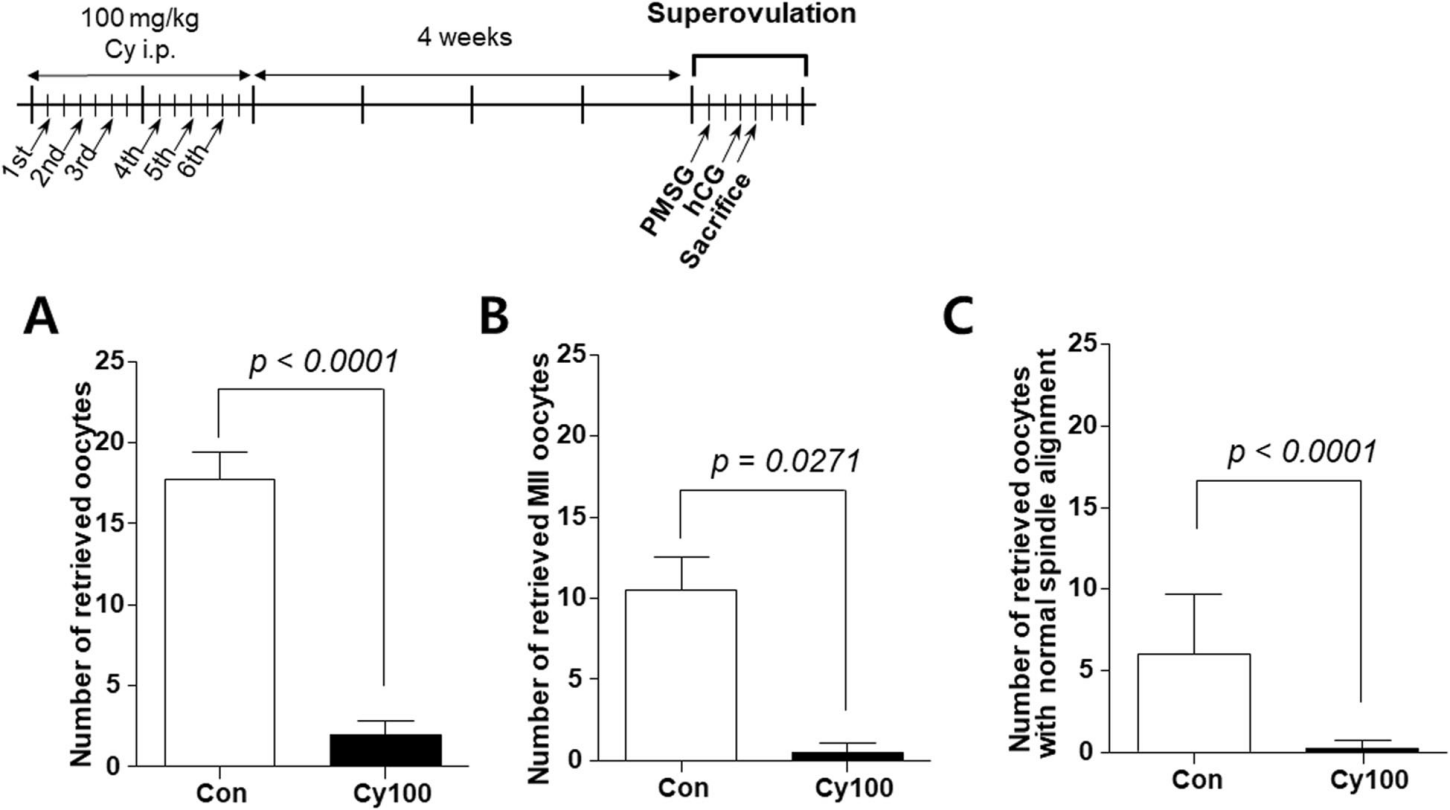
Quality of retrieved oocytes 4 weeks after Cy exposure Mice were injected with Cy (*n* = 4) or saline (*n* = 4) for 2 weeks, then housed for 4 weeks. The mice were superovulated and (**a**) total and (**b**) MII retrieved oocytes were quantified. The oocytes were immunostained for α-tubulin to assess spindle and chromosome alignment (**c**). Data represent the mean ± standard deviation. Statistical analysis was performed by Student’s *t*-test

As expected, the total number of retrieved oocytes and number that matured to metaphase II (MII) were both significantly decreased (Fig. 3a and b, *p* < 0.05). The MII oocytes of Cy-injected mice displayed increased chromosomal abnormalities and spindle misalignments compared to control oocytes (Fig. 3c, *p* < 0.001).

**Fig. 4:**
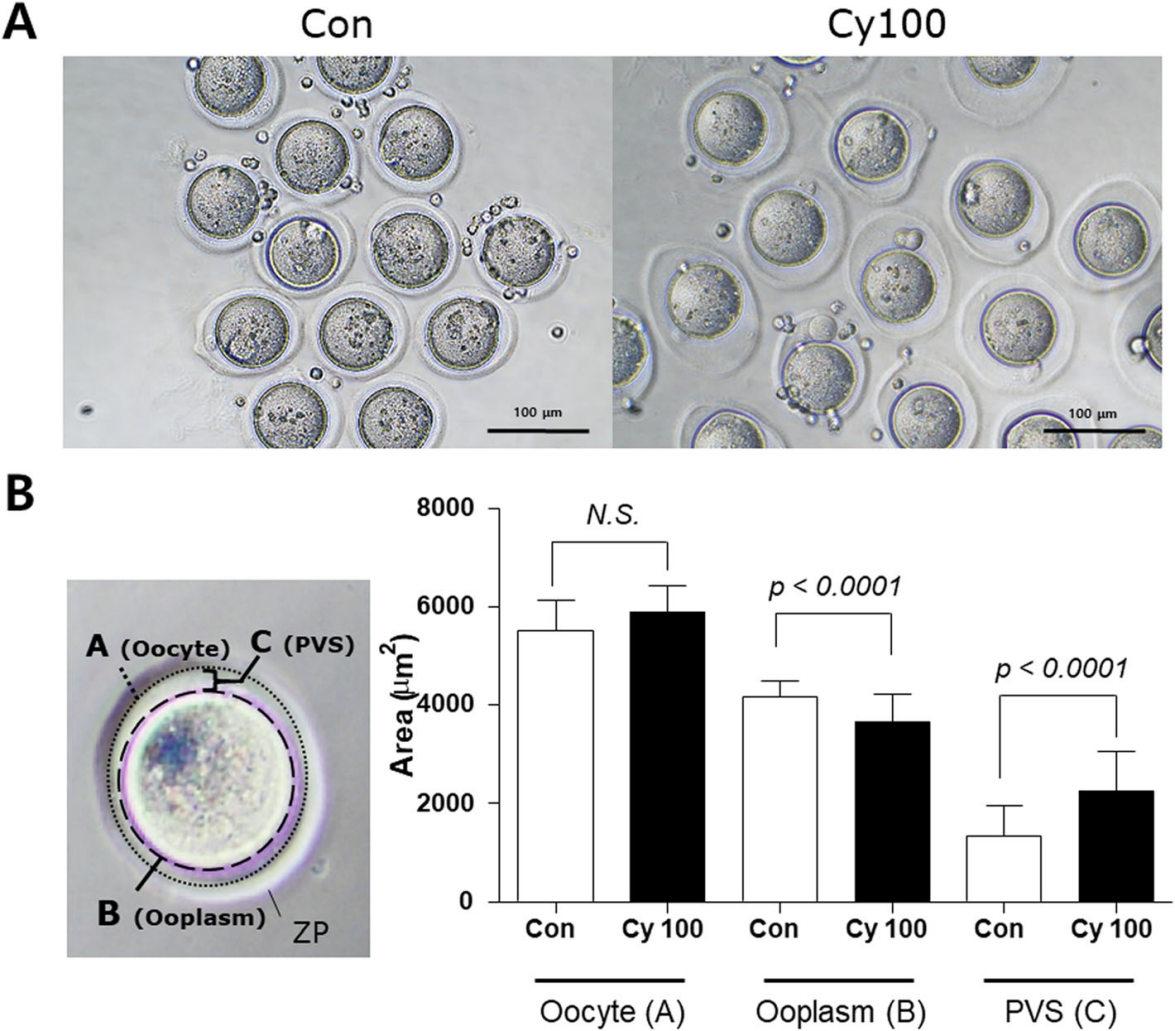
Retrieved oocytes 4 weeks after Cy exposure Mice were injected with Cy (*n* = 4) or saline (*n* = 4) for 2 weeks, and then housed for 4 weeks. **a** Mice were superovulated and the retrieved oocytes were observed. **b** Oocyte, ooplasm, and PVS were compared between the control and Cy-treated groups. Data represent the mean ± standard deviation. Statistical analysis was performed by Student’s *t*-test

Analysis of the morphology of Cy-treated oocytes revealed that the surrounding zona pellucida (ZP) was loosely compacted and less uniformly shaped than that in control oocytes (Fig. 4a). The area of the ooplasm was decreased in Cy-treated and control oocytes with a significantly increased PVS (Fig. 4b, *p* < 0.0001). Abnormal PVS morphology is a negative indicator of an oocyte’s developmental potential and has been linked to lower fertilization rates [19, 20]. These results indicate that even after chemotherapy ends, residual Cy metabolites or surviving follicles with damaged granulosa cells can impair oocyte viability and quality in response to superovulation.

### Gene expression is continuously altered in response to repeated cy exposure

**Fig. 5:**
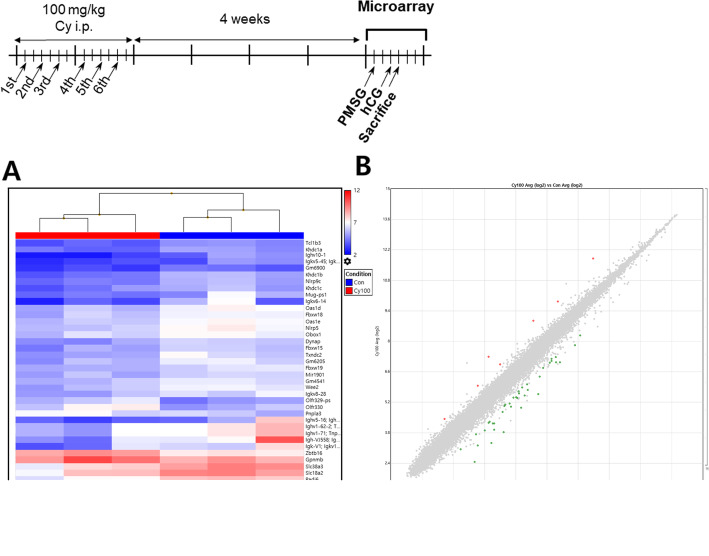
Hierarchical clustering and analysis of DEGs Ovaries from mice injected with Cy (*n* = 3) or saline (*n* = 3) were used for transcriptomic analysis. Microarrays were performed to compare ovarian gene expression 4 weeks after Cy exposure to that in control mice. **a** Hierarchical clustering among microRNA expression profiles. **b** Significantly upregulated and downregulated genes are shown as green and red dots, respectively

We next performed microarray experiments to analyze and compare the RNA expression patterns in ovaries from Cy- or saline-injected mice. Hierarchical clustering analysis revealed marked differences among the two mouse groups (Fig. 5a). Of the 41,345 genes detected, 46 were significantly different between the two groups (Fig. 5b). Of these, seven genes (15.2%) were upregulated and 39 genes (84.8%) were downregulated in Cy-injected mice compared with control mice (Tables 1 and 2). Interestingly, the expression profiles of genes associated with fertilization and ovarian follicle development, such as ZP glycoprotein 2 and 3 (*Zp2* and *Zp3*); solute carrier family 18, member 2 (*Slc18a2*); WEE1 homolog 2 (*Wee2*); NLR family, pyrin domain containing 5 (*Nlrp5*) and 2′-5′-oligoadenylate synthetase 1d and 1e (*Oas1d* and *Oas1e*), were changed. GO analysis revealed that in molecular functions, the DEGs were enriched in protein binding, acrosin binding, and 2′5′-oligoadenylate synthetase activity (Table 3). In biological process, enriched GO terms included ovarian follicle development, oogenesis, binding of sperm to ZP, and immune response (Table 3). For cellular components, GO analysis revealed that the DEGs were enriched in GO terms such as cytoplasm, secretory granule, extracellular region, and matrix (Table 3).

**Table 1:** Upregulated genes in Cy-exposed mice

Fold Change	*p*-value	Gene Symbol	Description
3.76	0.0128	*Olfr330*	Olfactory receptor 330
3.33	0.0014	*Stc1*	Stanniocalcin 1
2.97	1.40E-05	*Zbtb16*	zinc finger and BTB domain containing 16
2.53	0.0182	*Gpnmb*	Glycoprotein (transmembrane) nmb
2.08	0.0068	*Olfr329-ps*	Olfactory receptor 329, pseudogene
2.07	0.0298	*Pnpla3*	Patatin-like phospholipase domain containing 3
2.04	0.0024	*Gm24078*	Predicted gene, 24,078

Genes were significantly upregulated in Cy-injected mice when they displayed > 2.0-fold expression compared to in the control (*p* < 0.05)

**Table 2:** Downregulated genes in Cy-exposed mice

Fold Change	*p*-value	Gene Symbol	Description
4.92	0.0009	Ighv10–1	Immunoglobulin heavy variable 10–1
4.71	0.0402	Igk-V1	Immunoglobulin kappa chain variable 1
4.49	0.0204	Igkv6–14	Immunoglobulin kappa variable 6–14
4.05	0.0095	Ighv1–62-2	Immunoglobulin heavy variable 1–62-2
4.05	0.0095	Ighv1–71	Immunoglobulin heavy variable 1–71
3.71	0.0235	Igh-VJ558	Immunoglobulin heavy chain (J558 family)
3.25	0.0402	Ighv5–16	Immunoglobulin heavy variable 5–16
3.19	0.0184	Igkv5–45	Immunoglobulin heavy chain variable 5–45
2.93	0.0003	Nlrp14	NLR family, pyrin domain containing 14
2.79	0.0003	Oas1d	2–5 oligoadenylate synthetase 1D
2.70	0.0009	Slc38a3	solute carrier family 38, member 3
2.65	0.0203	Mug-ps1	Murinoglobulin, pseudogene 1
2.63	0.0049	Igkv8–28	Immunoglobulin kappa variable 8–28
2.59	3.59E-05	Zp2	zona pellucida glycoprotein 2
2.56	0.0001	Nlrp5	NLR family, pyrin domain containing 5
2.54	0.0002	Khdc1b	KH domain containing 1B
2.45	0.0009	Khdc1a	KH domain containing 1A
2.45	0.0266	n-R5s71	nuclear encoded rRNA 5S 71 [Source:MGI Symbol;Acc:MGI:4421916]
2.41	0.0056	Gm6205	predicted gene 6205
2.38	0.0007	Dynap	dynactin associated protein
2.31	0.0048	Slc18a2	solute carrier family 18 (vesicular monoamine), member 2
2.27	0.0023	Gm4541	Predicted gene 4541
2.25	0.0025	Txndc2	Thioredoxin domain containing 2
2.2	0.0021	Wee2	WEE1 homolog 2
2.19	0.0004	Nlrp9c	NLR family, pyrin domain containing 9C
2.18	0.165	Khdc1c	KH domain containing 1C
2.16	0.0003	Padi6	peptidyl arginine deiminase, type VI
2.15	8.81E-05	Gm1965	predicted gene 1965
2.14	0.005	Obox1	Oocyte specific homeobox 1
2.14	0.007	Tcl1b1	T cell leukemia/lymphoma 1B, 1
2.12	0.0002	Tcl1b3	T cell leukemia/lymphoma 1B, 3
2.12	0.0036	Gm6900	Predicted gene 6900 [Source:MGI Symbol;Acc:MGI:3645052]
2.11	0.0003	Fbxw19	F-box and WD-40 domain protein 19
2.08	0.0082	Fbxw15	F-box and WD-40 domain protein 15
2.07	0.0193	Ighv1–62-1	Immunoglobulin heavy variable 1–62-1
2.05	0.0025	Fbxw18	F-box and WD-40 domain protein 18
2.05	0.0084	Mir1901	microRNA 1901
2.02	0.0003	Zp3	zona pellucida glycoprotein 3
2.31	0.0053	Oas1e	2–5 oligoadenylate synthetase 1E

Genes were significantly downregulated in Cy-injected mice when they displayed > 2.0-fold differences compared to the control (*p* < 0.05)

**Table 3:** Functional annotation of differentially expressed genes

Category	Term	EASE Score	Count
GOTERM_CC_DIRECT	GO:0030141 ~ secretory granule	0.0001631	4
GOTERM_BP_DIRECT	GO:0001541 ~ ovarian follicle development	0.0017886	3
GOTERM_CC_DIRECT	GO:0005737 ~ cytoplasm	0.0021057	14
GOTERM_MF_DIRECT	GO:0032190 ~ acrosin binding	0.0029206	2
GOTERM_MF_DIRECT	GO:0005515 ~ protein binding	0.0079809	10
GOTERM_MF_DIRECT	GO:0001730 ~ 2′-5′-oligoadenylate synthetase activity	0.0106698	2
GOTERM_BP_DIRECT	GO:0006828 ~ manganese ion transport	0.0164696	2
GOTERM_CC_DIRECT	GO:0005576 ~ extracellular region	0.0225659	6
GOTERM_BP_DIRECT	GO:0045893 ~ positive regulation of transcription, DNA-templated	0.0244882	4
GOTERM_CC_DIRECT	GO:0005771 ~ multivesicular body	0.0276672	2
GOTERM_CC_DIRECT	GO:0031012 ~ extracellular matrix	0.032228	3
GOTERM_BP_DIRECT	GO:0006955 ~ immune response	0.0358449	3
GOTERM_CC_DIRECT	GO:0005578 ~ proteinaceous extracellular matrix	0.0367763	3
GOTERM_BP_DIRECT	GO:0048477 ~ oogenesis	0.0369613	2
GOTERM_BP_DIRECT	GO:0007339 ~ binding of sperm to zona pellucida	0.0390946	2

*GO* Gene ontology and *KEGG* Kyoto Encyclopedia of Genes and Genomes analysis was performed to identify potential functions of the differentially expressed genes in the *MF* molecular function, *BP* biological process, and *CC* cellular component categories

## Discussion

Currently, young women diagnosed with cancer have a greater chance of long-term survival than ever before. However, successful survivorship includes maintaining a high quality of life after cancer diagnosis and treatment [21], and lifesaving treatments such as chemotherapy, radiation, and surgery can impact survivors by impairing their reproductive and endocrine health. Patients exposed to several months of rigorous chemotherapy can suffer from infertility or premature ovarian failure. In addition, patients who retain fertility after cancer therapy have increased risk factors for fetal and maternal complications during subsequent pregnancies [22]. As fertility issues in cancer survivorship have become increasingly important, additional studies are needed to evaluate these effects. Most studies examining cancer therapy-related infertility have used mouse models induced by single or short-term exposure to anticancer agents, which is typically not performed in the clinic [3]. To consider the pathology of chronic ovarian impairment in young cancer survivors, we repeatedly administered Cy and analyzed physiological conditions at 4 weeks after treatment completion.

Cy exposure leads to deterioration of oocyte quality [23, 24]. Koike et al. reported a decrease in the number of retrieved oocytes, whereas the rates of fertilization and blastocyst development were similar compared to those in controls at 2 weeks after single 400 mg/kg Cy administration in mice [25]. However, the mouse model following single exposure of Cy did not reflect the clinical situation regarding the extent of damage to the follicle and oocyte quality in oocytes and embryos after rigorous Cy treatment.

Interestingly, both follicles and oocytes were susceptible to Cy-induced damage even after the 4 weeks had passed, allowing sufficient time for the generation of new preovulatory follicles [13]. Only a small number of oocytes were retrieved, which had phenotypic indications of low fertilization potential. This leads to poor reproductive outcomes such as a high risk of non-viable fetuses and malformation at 4 weeks after Cy exposure [26].

There is limited information regarding the effects of Cy in the ovaries. Cy is thought to act as a direct ovotoxin that destroys dormant primordial follicles and activates quiescent primordial follicles by inducing apoptosis in pregranulosa cells and oocytes [27]. Cy exposure also generates increased reactive oxygen species in oocytes, resulting in mitochondrial dysfunction and disrupting the meiotic spindle [23, 28].

To examine whether the adverse effects of rigorous chemotherapy persisted after cancer treatment ends, we performed microarray and bioinformatic analyses on ovaries 4 weeks after Cy exposure. In the microarray data, seven genes were found to be upregulated. Of them, Zbtb1 is a member of the Kr*ü*eppel C2H2-type zinc-finger protein family and regulated by the PI3K/PTEN/AKT pathway, which has a critical role in regulating dormancy and initial primordial follicle activation [29]. However, exposure to Cy disturbs this balance by destroying growing follicles or activating the PI3K/PTEN/Akt pathway, causing reservoir “burnout” [30, 31].

A total of 39 downregulated DEGs associated with folliculogenesis and oogenesis are involved in the prolonged effects of repeated Cy treatment. WEE2, one of the oocyte-specific kinase, is responsible for the follicular development, oocyte meiotic regulation, and fertilization in humans and mice [32, 33]. Reduced WEE2 levels induce fertilization failure and abnormal blastocyst formation [34]. Slc18a2 is highly expressed in granulosa cells of growing follicles and its downregulation indicates that granulosa cells were damaged by Cy exposure [35]. Additionally, expression of *OAS1D*, which regulates the translational regulator of newborn ovary homeobox gene (*Nobox*), was decreased, leading to rapid follicle loss after birth [36]. Taken together, altered gene expression continuously impaired ovarian follicle development even after Cy exposure was complete.

The DEGs also included oocyte-specific genes associated with fertilization, including *Zp1*, *2*, and *3*, which are critical for proper organization of the ZP surrounding oocytes and showed decreased expression 4 weeks after Cy exposure. Consistent with the microarray data, morphological observation revealed a loosely compacted ZP and enlarged PVS, which can affect fertility [37].

The microarray data indicate that genetic regulation in ovaries remains impaired 4 weeks after repeated Cy exposure. We found that impaired follicular growth correlated with oocyte abnormalities caused by rigorous Cy treatment. However, in our study, we did not determine whether these abnormal oocytes are directly caused by Cy and/or indirectly through other cells such as granulosa cells, and assessment of the fertilization potential of Cy-damaged oocytes requires further studies. In recent decades, several studies have been conducted to develop strategies for protecting fertility from the effects of chemotherapeutic drugs, but they have been performed during chemotherapy in animal models [38]. Our results suggest that, for young cancer survivors, persistent treatment is required to prevent chronic damage to the ovaries after chemotherapy ends.

## Conclusion

We found that ovarian cell damage induced by repeated Cy treatment continuously alters the expression of genes associated with fertility and has persistent effects on ovarian function, resulting in diminished ovarian reserves even after the completion of chemotherapy. Studies to prevent chronic damage to the ovaries and/or restore their function are required to ensure fertility preservation in cancer survivors.

## Data Availability

All data generated and analyzed during this study are included in this article. The datasets generated and/or analysed during the current study are available from the corresponding author on reasonable request.

## Abbreviations

AMH: Anti-Müllerian hormone
Cy: Cyclophosphamide
DEGs: Differentially expressed genes
GO: Gene ontology
hCG: Human chorionic gonadotropin
KEGG: Kyoto Encyclopedia of Genes and Genomes
*Nobox*: Newborn ovary homeobox gene
*Nlrp5*: NLR family, pyrin domain containing 5
*Oas1d*: 2′-5′-oligoadenylate synthetase 1d
*Oas1e*: 2′-5′-oligoadenylate synthetase 1e
PVS: Perivitelline space
PMSG: Pregnant mare’s serum gonadotropin
*Slc18a2*: Solute carrier family 18, member 2
*Wee2*: WEE1 homolog 2
